# Systematic Review of Tools and Approaches for Evaluating the Transferability of Health Technology Assessments Across Different Jurisdictions

**DOI:** 10.34172/ijhpm.8218

**Published:** 2024-10-02

**Authors:** Elham Ahmadnezhad, Mehrnaz Kheirandish, Ali Akbari-Sari, Arash Rashidian

**Affiliations:** ^1^Health System Observatory Secretariat, National Institute of Health Research, Tehran University of Medical Sciences, Tehran, Iran.; ^2^Department of Science, Information and Dissemination, WHO Regional Office for the Eastern Mediterranean, Cairo, Egypt.; ^3^Department of Health Management and Economics, School of Public Health, Tehran University of Medical Sciences, Tehran, Iran.

**Keywords:** Transferability, Health Technology Assessment, Evidence-Informed Policy-Making, Economic Evaluation, Adaptability, Generalizability

## Abstract

**Background:**

This study aims to review tools that have been developed for the transferability of health technology assessment (HTA) information to different countries. HTA is increasingly being used as a tool in health policy decision-making, but its complexity and lack of local expertise have limited its usage in many countries. The World Health Organization (WHO) has taken measures to encourage countries to conduct and use HTA, including through resolutions from the Eastern Mediterranean (EM) Regional Committee in 2019. However, due to limitations in national technical capacities, there is a need to adapt HTA information from other settings to fit the specific context of each country. Therefore, this study aims to systematically review the tools that have been developed for HTA transferability and assess their strengths and limitations.

**Methods:**

The systematic review included studies that introduced tools, methods, and frameworks for transferability of HTA information across jurisdictions. Databases such as MEDLINE, EMBASE, Cochrane Library, Epistemonikos, Web of Science, health economic database, Scopus, and Google Scholar were searched, along with relevant bibliographies. The data was extracted and synthesized using both tabulation and narrative approaches. The evaluation of the tools involved assessing various criteria, such as user-friendliness, efficiency in screening, and considerations regarding transferability factors.

**Results:**

A total of 10 375 documents were evaluated, resulting in 17 studies that met the inclusion criteria. These 17 studies consisted of 13 newly developed tools/methods that were appraised. The majority of the models were checklists, with only a few deemed suitable for full HTA. Three models have been validated through published studies, but there is no evidence of utilization in the countries of the EM region.

**Conclusion:**

While the existing tools provide valuable resources for evaluating transferability, there remains a need for a more comprehensive tool to support decision-makers in low-resource settings considering country context and capacity.

## Background

 In recent years, the goal of universal health coverage (UHC) has emerged as a key priority for health system strengthening.^1^ A critical factor in achieving UHC is the cost of healthcare, which plays a significant role.^2^ Health technology assessment (HTA) has emerged as a tool to support informed decision-making and efficient cost allocation towards the goal of UHC.^2^ HTA is recognized as one of the six decision-support pillars in the context of the World Health Organization (WHO) Regional Office for Eastern Mediterranean (EM) and is included in the regional action plan for evidence-informed policy-making (EIPM) as a strategic domain for the development of national capacity.^3^

 Interest in HTA has grown, particularly in the EM region, and this trend is expected to escalate following the COVID-19 pandemic, similar to other settings.^4-7^ In recent decades, many high-income countries have developed their own HTA systems and published numerous HTA reports.^8^ However, low- and middle-income countries (LMICs) including EM countries may have limited capacity, expertise, and time to establish a national HTA program to perform the necessary HTA studies.^4,6,8,9^

 A question of interest is whether these countries can use the results of HTA studies performed by other countries with different contexts, and to what extent these results are transferable. Over the past few decades, the issue of HTA transferability^[1]^ has been explored.^6,7,10,11^ Two reviews have been published, one in 2011 and another in 2022, that assess the tools developed for the transferability of HTA information.^7,10^ The focus of the latter review was the applicability and practical use of existing tools in LMICs.^6^ These two well-described reviews highlight the importance of the applicability of HTA transferability tools, taking into account their limitations, especially in LMICs.

 Given that most of the member states of the EM region have not yet established their own national HTA programs, this study aims to review the available tools and methods that can support the transferability of HTA information from studies conducted in other regions to the EM region countries.

## Methods

 A systematic review was conducted from March 15, 2021 to April 15, 2021 to identify tools and methods for assessing the transferability of HTA information across jurisdictions. The main source of literature was journal articles, but all relevant sources including books, reports, theses, and conference papers were considered. The databases were searched from 1995 onwards.

###  Inclusion and Exclusion Criteria 

####  The Inclusion Criteria

- All tools, methods, checklists and frameworks developed for assessing the transferability of HTA information across jurisdiction.
- All English papers and reports published from 1995 onwards were included.

####  The Exclusion Criteria

- Non-English language papers (full text).
- Studies that discuss other subjects related to HTA and do not introduce any tools or methods for HTA transferability.

###  Searched Databases 

 The review searched various health-related databases including MEDLINE, EMBASE, Cochrane Library, Epistemonikos, and Web of Science. Additionally, specialized health economics databases such as EconLit, Economic Working Papers Database (RePEC: idea), Health Economic Evaluation Database (HEED) and the National Health Service Economic Evaluation Database (NHS EED) were also searched. The search was optimized by using both Scopus and Google Scholar. The search strategy was comprehensive, combining electronic database searches with hand searches of relevant bibliographies. All references were managed using an EndNote version X7 database manager, with duplicates removed and remaining references checked manually.

###  Screening and Data Extraction 

 The screening process involved a comprehensive evaluation of all relevant literature. Two researchers independently performed a thorough review of the titles and abstracts of all identified articles. Subsequently, the full text of each eligible article was obtained for further assessment. The final selection of articles was based on their alignment with the predefined inclusion and exclusion criteria. Apart from the general characteristics of each document (title, publication date, authors, …), all included documents were analyzed into 12 distinct elements that served as the basis for evaluating and synthesizing the findings of the studies.

###  Evaluation Criteria

 The development of the evaluation criteria was carried out through a meticulous process, which included the following steps:

1. Literature review: The process began with an extensive review of relevant literature, focusing on transferability tools, HTA, and economic evaluation methodologies. This step aimed to identify key elements and factors that should be considered when assessing the transferability of HTA information across different jurisdictions.
2. Expert consultation: A panel of experts comprising specialists in HTA and economic evaluation was convened for consultation. The study team presented the preliminary set of evaluation criteria compiled from the literature review to the expert panel. The experts engaged in discussions, providing valuable insights, feedback, and suggestions to refine and enhance the criteria.
3. Feedback analysis and synthesis: The input and perspectives shared by the expert panel were carefully analyzed and synthesized by the study team. The feedback received was used to make adjustments to the initial set of criteria, ensuring clarity, relevance, and comprehensiveness.
4. Iterative discussions and revisions: Following the feedback analysis, the study team engaged in iterative discussions and revisions to refine the evaluation criteria further. This process involved addressing any identified gaps, removing redundant elements, and ensuring that the criteria were both comprehensive and concise.
5. Finalization of evaluation criteria: After several rounds of discussions and revisions, the final set of evaluation criteria was established. This refined set of criteria provided a comprehensive framework for assessing transferability tools, taking into account various dimensions such as usability, consideration of relevant factors, validation methods, and applicability across different contexts.

In this study, we employed several elements to compare the transferability models:

1. Ease of use: Refers to the simplicity and user-friendliness of the tool. This criterion assesses how intuitive and straightforward the tool is for users to navigate and utilize effectively. A user-friendly tool should have clear instructions, a logical interface, and minimal complexity, allowing users to easily understand its functionalities and apply it without extensive training or technical expertise. Features such as user-friendly interfaces, clear instructions, and intuitive navigation contribute to the overall user experience, making it easier for individuals to adopt and use the tool efficiently. Evaluating user-friendliness involves considering factors such as the clarity of instructions, accessibility of features, and overall usability of the tool to ensure that it can be effectively utilized by a wide range of users, regardless of their level of experience or expertise.
2. Rapid screening criteria: Refers to the presence of quick evaluation criteria for preliminary assessment of transferability.
3. Factors affecting transferability: Refers to the tool’s ability to consider various factors impacting transferability. These factors may include differences in healthcare systems, patient demographics, cultural norms, regulatory environments, and economic conditions. A robust tool for assessing transferability should systematically address these diverse factors to provide a thorough assessment of the applicability of HTA findings in new contexts.
4. Utilization across fields: This criterion evaluates the extent to which the tool can be applied across various domains within HTA and economic evaluation. It encompasses the breadth of HTA approaches employed, such as partial or full assessments, as well as the range of economic evaluation methods utilized, which may include modeling-based analyses and trial-based evaluations. Essentially, it assesses the tool’s versatility and applicability across different methodologies and areas within the realm of HTA and economic evaluation.
5. Testing and field validation: Refers to thorough testing during development and in various fields by developer(s). “Thorough” in this context refers to conducting comprehensive testing of the developed tool in real-world settings after its development.
6. Development process documentation: Refers to transparent documentation of the tool creation process.
7. Tool type: Refers to the format or structure of the tool, eg, checklist, model, chart, or framework.
8. Peer-review: Refers to the evaluation of the tool by field experts for validity and reliability.
9. Scoring method (if applicable): Refers to the scoring system used to assess transferability, if applicable.
10. Organization endorsement: Refers to recognition and endorsement by a professional organization or governing body. Organizational endorsement implies that an organization, potentially related to HTA or another relevant field, has officially recognized and supported the tool. This endorsement signifies that the introduced tool has likely undergone rigorous stages of development, such as review of the developed protocol and evaluation of results. It is important to note that while some tools may have been independently developed by a team, others may have been commissioned by organizations. However, the use of the tool itself is not necessarily integrated into the formal HTA process, although it may be utilized within such contexts depending on the organization’s practices and policies.
11. Transferability/generalizability assessment: Refers to the tool’s ability to evaluate and account for various factors that influence transferability and generalizability, which are essential for the successful adaptation of HTA information across different jurisdictions^[2]^.
12. Validation through published studies: This criterion assesses the tool’s validation and reliability through testing and comparison with other published studies. Unlike Criterion 5 (Testing and field validation), which focuses on the tool’s usage by its developer, Criterion 12 examines its adoption by external entities or users.

These criteria were analyzed solely based on their presence or absence, without assigning any weight to individual criteria.

###  Search Algorithm 

 After screening and hand searching relevant review papers, 10 375 documents remained in the bibliographic database and 1744 in the health economic databases. After removing duplicates in the health economic databases, 566 documents were available, all of which were also available in the bibliographic databases. The Preferred Reporting Items for Systematic Reviews and Meta-Analyses (PRISMA) algorithm was drawn only for the bibliographic databases. At the final step, 17 documents satisfied the inclusion criteria and were taken into consideration (Figure). The search strategy is provided in Supplementary file 1.

**Figure:**
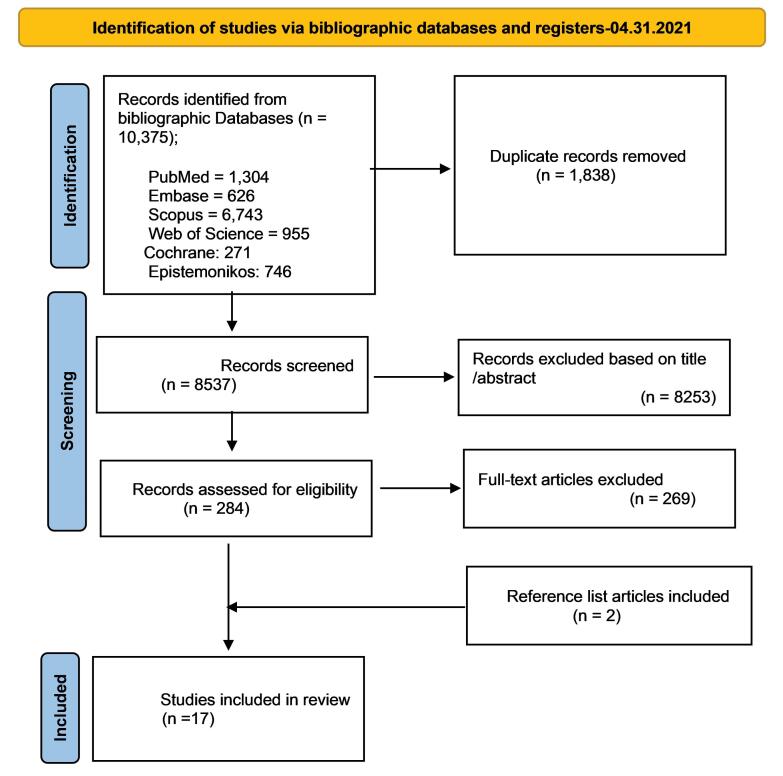
Search Algorithm in the Bibliographic Databases.

## Results

 Among the total number of reviewed documents (10 375), 17 studies presented various tools, checklists, and indicators for assessing the transferability of HTA reports. A list of these studies is presented in Table 1.^11-28^

**Table 1:** The Documents Which Introduced Tool, Method, Checklist, and Framework (Type) on the Health Technology Assessment Transferability

**Name of Developer**	**Date of Released**	**Type**	**Term Used**	**Associated Organization**
Heyland^12^	1996	Criteria/Checklist	Generalizability	McMaster University
Späth^13^	1999	Indicator/Checklist	Transferability	None
Greiner^14^	2000	Checklist	Transferability	None
Welte^15^	2004	Framework	Transferability	None
Sculpher^16^	2004	Checklist	Generalizability	None
Boulenger^17^ (EURONHEED)	2005	Checklist	Transferability/Adaptation	York University
Drummond^18^	2005	The same with Sculpher,^16^ which is published separately		
Urdahl^19^	2006	Checklist	Generalizability	None
NIHR-Adat Toolkit^11^ (EURONHEED2)	2009	The same with Boulenger^17^ (EURONHEED), which is updated		
ISPOR-Chart^22^	2009	Chart	Transferability	ISPOR
Antonanzas^23^	2009	Index	Transferability	None
Chase^24^	2009	Toolkit	Transferability/Adaptation	NIHR-UK
Turner^25^	2009	The same with Chase,^24^ which is published separately		
NHS-Adapt Toolkit^11^	2011	The same with Chase,^24^ which is updated.		
Mulline^26^	2014	Checklist	Not specified	None
MEEP project^27^	2014	Method	Not specified	Bill and Melinda Foundation
SEED tool^28^	2019	Checklist	Transferability	None

Abbreviations: ISPOR, International Society for Pharmacoeconomics and Outcomes Research; SEED, Systematic thinking for evidence based and efficient decision-making; NIHR, National Institute for Health and Care Research; MEEP, Methods for Economic Evaluation Project; NHS, National Health Service; EURONHEED, EUROpean Network of Health Economics Evaluation Databases.

 The first model aimed at assessing the transferability was released in 1996, with the most recent one being introduced in 2019. Out of the 17 documents reviewed, 13 can be considered as new tools, frameworks, charts, checklists, or models. Three studies have provided updates or modifications to previously developed tools and methods. One of the studies, the Methods for Economic Evaluation Project (MEEP) project, is not a tool but rather a reference case that refers to the transferability of HTA or economic evaluation and has been included in this review.

 The tools were assessed based on the proposed evaluation criteria as follows (Table 2):

- Ease of use: Three models were identified as easy to use.
- Rapid screening criteria: Nine models incorporate rapid screening criteria.
- Factors affecting transferability: Six models consider the majority of relevant factors.
- Utilization across fields: Only two models are suitable for comprehensive HTA.
- Testing and field validation: Ten models have been tested in one or more case studies.
- Development process documentation: Eight models offer information on their development process.
- Tool type: The majority of models are checklists.
- Peer-review: Seven models have undergone peer-review.
- Scoring method: Three models include a scoring system.
- Organization endorsement: Six models have received endorsement from an organization.
- Transferability/generalizability assessment: Three models lack any assessment in this regard.
- Validation through published studies: Three models have been validated through published studies. (No evidence was found indicating the utilization of these tools in the countries of the EM region).

**Table 2:** Comparison of the Reviewed Tools

**Name**	**Ease of Use**	**Rapid Screening Criteria**	**Consideration of Most Factors Affecting Transferability**	**Utilized in Various Fields**	**Testing and Field Validation**	**Development Process Documentation**	**Type of Tool**	**Peer-Review of the Checklist**	**Type of Scoring (if applicable)**	**Endorsement by an Organization**	**Assessment of Transferability/** **Generalizability Factors**	**Validation through Published Studies**
Heyland (12)	Yes	Yes	No	Economic evaluation (modelling)	Yes (intensive care unit)	No	Checklist	No	No explicit scoring	No	Patient factor, cost and discount rate (included)-resource use and base-line risk (not included)	No
Späth (13)	Yes	Yes	No	Economic evaluation (modelling)	Yes (Adjuvant therapy in women with breast cancer)	No	Checklist	No	No explicit scoring	No	Patient factor, cost, health outcome data, discount rate and resource use	No
Greiner (14)	Yes	Yes	No	Economic evaluation (modelling)	No	No	Checklist	No	No explicit scoring	No	Not defined	No
Welte (15)	Relatively (answers are quite subjective)	Yes	Yes	Full HTA (trial and modelling based)	Yes (For three cases: (1) Percutaneous transluminal coronary angioplasty for coronary heart disease in Germany; (2) Vaccine candidate’s cost-effectiveness in Dutch; and (3) Chlamydial screening programme in Denmark)	Yes	Checklist	Yes	Low to high	No	14 Factors in 3 categories (1) Methodologic; (2) Healthcare system; and (3) Patient	Yes
Sculpher (16)	Yes	Yes	No	Economic evaluation (modelling and trial based)	Yes (Osteoporosis)	No	Checklist	No	No explicit scoring	NHS	Not defined	No
Boulenger (17) (EURONHEED)	Relatively	No	Yes	Economic evaluation (modelling) and full HTA	Yes (>27 studies in different fields)	Yes	Checklist and quantitative index based	Yes	Explicit scoring (o, 0.5. 1)	NIHR (UK)	Health technology, setting, patient characteristics, health benefit, cost, discount rate, patient	Yes
Urdahl (19)	Yes	Yes	No (Only four questions to answer)	Economic evaluation (modelling and trial based)	Yes (Osteoporosis)	No	Checklist	No	No explicit scoring	NHS	Not defined	No
ISPOR-Chart (22)	Yes	Yes	No	Economic evaluation (modelling and trial based)	Yes (Pharmaeconomic guideline)	Yes	Checklist	Yes	No explicit scoring	ISPOR	Cost, discount rate, resources use	Yes
Antonanzas (23)	Several elements of study are addressed and weights and interpreting score are difficult and it takes a long list of questions	No	Yes	Economic evaluation (modelling and trial based)	Yes (27 Spanish studies)	Yes	Quantitative index based	Yes	Index with weights	No	Cost, discount rate, resources use	No
NIHR-Adapt toolkit (11) (2)	Several elements of study are addressed, interpreting score are difficult and it takes a long list of questions (need 5-day for using the main part)	Yes	Yes	Focus on HTA reports	Yes	Yes	Checklist	Yes	No explicit score	NHS	The main part of the toolkit can be used only to adapt information and/or data contained within an HTA report that includes one or more of these five domains. Currently, this toolkit would not enable the user to adapt information and/or data on legal, social or ethical aspects	Yes
Mulline (26)	Several elements of study are answers and answers are subjective	No	Yes	Pharmacoeconomic models (specific for model adaptation)	No	Yes	Checklist	No	No explicit score	No	Cost, discount rate, resources use	No
MEEP project (27)	Several activities should be done	No	No	Economic evaluation	No	Yes	Checklist	No	No explicit score	Bill and Melinda Gates Foundation, NICE International, the Health Intervention and Technology Assessment Program (Thailand), and the University of York, Centre for Health Economics	Not defined	No
SEED tool (28)	Several elements of study are answers and answers are subjective	Yes	Yes	Economic evaluation	Yes (NCD field)	Yes	Checklist	No	No explicit score	No	Baseline risk, cost, discount rate, resources use, treatment effect, health state preference weight	No

Abbreviations: ISPOR, International Society for Pharmacoeconomics and Outcomes Research; SEED, Systematic thinking for evidence based and efficient decision-making; NIHR, National Institute for Health and Care Research; MEEP, Methods for Economic Evaluation Project; NHS, National Health Service; EURONHEED, EUROpean Network of Health Economics Evaluation Databases; NICE, National Institute for Health and Care Excellence; HTA, health technology assessment; NCD, non-communicable disease.

## Discussion

 This systematic review was conducted to evaluate the tools developed for the transferability of HTA information. Findings revealed that several tools and methods have been established to assess the transferability of HTA information, each with its own strengths and limitations, and serving various purposes. Thirteen tools for HTA transferability were identified globally, with four of them (Welte, EUnet, European Network of Health Economics Evaluation Databases [EROUNHEED], and International Society for Pharmacoeconomics and Outcomes Research [ISPOR]) being utilized more frequently.^11,15,21,22^ No peer-reviewed literature documents the use of the remaining tools identified in this study, and no studies report their application in different settings. While all 13 tools are critiqued, only mentioned four frequently used tools are discussed in detail, excluding the developers and other settings.

 SEED tool conducted an evaluation of the present tools for transferability in HTA in 2019. The research underscores the critical factors that must be taken into account when transferring HTA and puts forward a decision-making framework that prioritizes local relevance with a focus on Best and Wasted Buys. The SEED tool is distinct in identifying the gaps and subsequently devising a framework and checklist to address them. Additionally, the investigation accentuates the factors that impact transferability, while abstaining from subjecting the content of the existing tools to a critical review.^28^

 Our review revealed that none of the tools were designed with the intention of filling any existing gaps or completing previous tools. The purpose for the development of these tools has not been stated as a means to address such gaps or complementarities in the existing tools. All domains of HTA or address all aspects of transferability issues identified in the review.

 A systematic review conducted in 2011 aimed to evaluate HTA transferability tools and found a significant variability in the approaches used for assessing transferability. The review did uncover an extensive checklist of factors, critical and noncritical, that could serve as a basis for a future consensus-based tool. However, the task of assigning appropriate weights to noncritical factors raises concerns about the feasibility and usefulness of developing a transferability score or index. The findings indicate that a comprehensive tool for analyzing all the factors affecting the transferability of HTA information from one setting to another remains yet to be developed.^10^ A subsequent scoping review conducted in 2022 reviewed 19 studies and found the EUnet HTA Adaptation Toolkit^11^ to be the most comprehensive among the methods and tools reviewed. Despite this, none of the identified tools fully encompass all domains of HTA or address all transferability issues.^7^

 The EUnet HTA Adaptation Toolkit,^11^ which was recommended in the 2022 review,^7^ has limitations in its application. Despite being developed to address the shortcomings of previous tools, it still requires subjective judgment in answering a significant number of questions. The tool aims to assess transferability, relevance, and reliability of HTA information but its complexity may hinder its use. It is general in nature and covers a variety of report types, and does not specifically address organizational factors such as legal, social, and ethical aspects or the transferability of diagnostic tests and screening technologies.

 A tool developed by Welte et al,^15^ has been widely used as a model for determining the transferability of HTA reports. The tool consists of a checklist with general and specific criteria for assessing transferability, but it does not fully consider factors such as the health system, country context, and the quality of reported results that may have a significant impact on the transferability of HTA information. The Welte model does not address the adaptation of HTA information to the context of a specific country or how to adapt HTA information in settings with limited resources. Since the evaluation is based on qualitative answers, the use of multidisciplinary team may be necessary in setting where the capacity for HTA is limited.

 The EROUNHEED tool^17,21^ is a transferability assessment tool consisting of two sections, that contains 42 questions aimed at evaluating the quality and generalizability of results in HTA. Although the tool is comprehensive, its application lacks clarity and its quantitative approach, which assigns scores between 0 and 100, may limit the validity of transferability evaluations. This is because some HTA studies that receive high scores may not be highly transferable due to factors that are not taken into consideration in the scoring system.^21^

 The final tool to be evaluated is the ISPOR transferability tool,^22^ which is based on the Welte model^15^ and shares its limitations. This tool involves a series of four questions for assessing transferability, however, its application may be complex for individuals who are unfamiliar with economic evaluation as it requires an understanding of various criteria and considerations. Additionally, some aspects of the tool rely on subjective judgments, leading to potential variations in results between different users. This complexity may make the use of the ISPOR tool challenging for those who are not well-versed in the field of economic evaluation.

 The aforementioned four tools^11,15,21,22^ have gained significant recognition and usage in the field of HTA and economic evaluation. However, despite their popularity, each of these tools has its own limitations, and a more comprehensive tool for the transferability of HTA reports and economic evaluation is still sought after.

 Across the array of reviewed tools, comprehensive attention is given to HTA information, covering biological or clinical data alongside aspects related to cost-effectiveness or economic value transfer. While transferring clinical information between settings is generally feasible, the process becomes notably complex when dealing with economic outcomes, presenting a recurrent challenge in these tools. Successfully transferring economic information demands meticulous methods and strategies. Therefore, future endeavors in tool development should prioritize addressing this complexity to bolster the transferability of economic discussions.

 Despite the limited presence of an established HTA system in most of the countries in the EM region, our literature review did not identify any peer-reviewed or gray literature indicating the implementation of transferability tools for HTA in the region.

 Our findings suggest that the development of a new and more appropriate tool is required to facilitate the integration of HTA information into EIPM in LMICs in the EM region may be a viable option. This assertion is supported by a recent review, which introduces a forward-looking model for HTA implementation in LMICs, proposing innovative HTA approaches for adoption. These approaches hold the potential to advance HTA in ways tailored to LMIC contexts, offering promise in improving healthcare decision-making beyond the conventional scope of determining service and medicine coverage.^7^ We also recognize the significance of real-world evidence and real-world data in shaping healthcare decision-making. The successful transferability of HTA findings hinges on contextualizing real-world evidence practices, which necessitates engaging multiple stakeholders and reaching consensus on data collection, sharing, and utilization. This emphasizes the importance of robust tools to evaluate HTA transferability, ensuring alignment with local healthcare contexts and decision-making procedures.

 This review focused solely on documents that explicitly introduced tools or methods for HTA transferability, omitting studies that applied these tools. Future research could enhance this review by evaluating the practical application of such tools. One notable limitation is that the literature search was conducted over two years ago, potentially excluding more recent studies. Thus, it’s essential to recognize and explore additional research published after the search period for a comprehensive understanding. Additionally, the appraisal elements used in this study were compiled by the authors, suggesting potential limitations in their inclusivity. However, the absence of a comprehensive critical appraisal tool for HTA transferability studies should also be acknowledged. Lastly, the restriction to English-language documents may have overlooked tools used in other languages and settings, underscoring a limitation in language inclusivity.

## Conclusion

 The purpose of this review was to evaluate the current tools utilized for transferability of HTA information to another setting and to identify any unaddressed limitations and shortcomings and aimed to identify areas where improvement is needed particularly for EM countries. The need for a more effective tool is increasingly important, especially with the increasing emphasis on using best evidence for decision-making processes. In order to develop a new tool, several key considerations must be taken into account. Firstly, the tool should consider a wider range of factors that may impact transferability. This will provide a more comprehensive understanding of the factors that can affect the successful transfer of HTA reports from one setting to another. Secondly, a method for evaluating the quality of HTA reports and papers should be incorporated into the tool. This is essential for ensuring the accuracy of results and for providing decision-makers with reliable information. Thirdly, contextual factors such as financial and applicability challenges, as well as any factors that may limit the contextualization of HTA reports, should also be taken into account. These considerations have not been addressed clearly in previous tools and are crucial for promoting the acceptance and adapting of HTA. These aspects have received limited attention in existing and is particularly relevant in regions (such as EM region) where HTA systems are still developing. The increasing focus on EIPM in the EM region shows the need for the development of a transferability tool addresses this limitation and cover the key criteria for contextualization of the result of the HTA studies from another countries to EM countries.

## Acknowledgements

 The authors would like to acknowledge the support provided by the WHO Regional Office for the EM region in conducting this study. The authors would also like to express their gratitude to Drs Alireza Olyaeemanesh, Mohammadreza Mobinizadeh, Zahra Gharib-Naseri, Marita Mohammadshai, Sommayeh Afshari, and Behzad Raii for their invaluable contributions in participating in the study review meetings.

## Ethical issues

 Not applicable.

## Conflicts of interest

 Authors declare that they have no conflicts of interest.

## Disclaimer

 The views expressed in this article are solely those of the authors and do not reflect the views, decisions, or policies of the WHO.

## Endnotes


^[1]^ In this paper, we use terms like transferability, generalizability, adoption, adaptation, portability, exchangeability, and extrapolation interchangeably to describe how analyses and results are applied across different jurisdictions in the fields of economic evaluation and HTA.
^[2]^ It is important to acknowledge that terms like transferability, generalizability, adaptability, and adaptation are frequently used interchangeably across literature. However, this paper specifically emphasizes transferability to ensure a comprehensive review of relevant literature. For more information, please refer to Supplementary file 1.

## Supplementary files

Supplementary file 1. Search Strategies in the Searched Databases.
