# Supplementary material for: Systematic Review of Tools and Approaches for Evaluating the Transferability of Health Technology Assessments Across Different Jurisdictions
Source: Int J Health Policy Manag. 2024 Oct 2;13:8218. doi: 10.34172/ijhpm.8218 (PMC11549564; doi:10.34172/ijhpm.8218)
Supplement: Supplementary file 1 — Search Strategies in the Searched Databases. [file ijhpm-13-8218-s001.pdf]

Article title: Systematic Review of Tools and Approaches for Evaluating the Transferability of Health Technology Assessments Across Different Jurisdictions

Journal name: International Journal of Health Policy and Management (IJHPM)

Authors' information: Elham Ahmadnezhad[1], Mehrnaz Kheirandish[2]*, Ali Akbari-Sari[3], Arash Rashidian[2]

[1]Health System Observatory Secretariat, National Institute of Health Research, Tehran University of Medical Sciences, Tehran, Iran.

[2]Department of Science, Information and Dissemination, WHO Regional Office for the Eastern Mediterranean, Cairo, Egypt.

[3]Department of Health Management and Economics, School of Public Health, Tehran University of Medical Sciences, Tehran, Iran.

***Correspondence to:** Mehrnaz Kheirandish; Email: kheirandishm@who.int

Citation: Ahmadnezhad E, Kheirandish M, Akbari-Sari A, Rashidian A. Systematic review of tools and approaches for evaluating the transferability of health technology assessments across different jurisdictions. Int J Health Policy Manag.2024;13:8218. doi:10.34172/ijhpm.8218

Supplementary file 1. Search Strategies in the Searched Databases

Date: 15 March-15 April 2021

Search strategies in all bibliographic databases

Data bases	Search strategy	NO. of documents
PubMed	((Technology Assessment Biomedical[tiab]) OR (health technology assessment[tiab]) OR (Cost-Benefit Analysis[tiab]) OR (economic evaluation analysis[tiab]) OR (cost-effectiveness Analysis[tiab]) OR (cost-utility Analysis[tiab]) OR (HTA[tiab]))<br>AND<br>((applicability[tiab]) OR (generalisability[tiab]) OR (generalizability [tiab]) OR (adaptability [tiab]) OR (adaptation[tiab]) OR (transferability[tiab]) OR (portability[tiab]) OR (adoption[tiab]) OR (exchangeability[tiab]) OR (Toolkit*[tiab]) OR (Checklist*[tiab]))	1304

Data bases	Search strategy	NO. of documents
**Scopus**	(TITLE-ABS(Technology Assessment Biomedical) OR TITLE-ABS(health technology assessment) OR TITLE-ABS(Cost-Benefit Analysis) OR TITLE-ABS(economic evaluation analysis) OR TITLE-ABS(cost-effectiveness Analysis) OR TITLE-ABS(cost-utility Analysis) OR TITLE-ABS(HTA)) AND (TITLE-ABS(applicability) OR TITLE-ABS(generalisability) OR TITLE-ABS(generalizability) OR TITLE-ABS(adaptability) OR TITLE-ABS(adaptation) OR TITLE-ABS(transferability) OR TITLE-ABS(portability) OR TITLE-ABS(adoption) OR TITLE-ABS(exchangeability) OR TITLE-ABS(Toolkit*) OR TITLE-ABS(Checklist*))	**6743**
**Web of science**	(ALL=(Technology Assessment Biomedical) OR ALL=(health technology assessment) OR ALL=(Cost-Benefit Analysis) OR ALL=(economic evaluation analysis) OR ALL=(cost-effectiveness Analysis) OR ALL=(cost-utility Analysis) OR ALL=(HTA)) AND (TI=(applicability) OR TI=(generalisability) OR TI=(generalizability) OR TI=(adaptability) OR TI=(adaptation) OR TI=(transferability) OR TI=(portability) OR TI=(adoption) OR TI=(exchangeability) OR TI=(Toolkit*) OR TI=(Checklist*))	**955**
**Embase**	((Technology Assessment Biomedical:ti,ab) OR (health technology assessment:ti,ab) OR (Cost-Benefit Analysis:ti,ab) OR (economic evaluation analysis:ti,ab) OR (cost-effectiveness Analysis:ti,ab) OR (cost-utility Analysis:ti,ab) OR (HTA:ti,ab)) AND ((applicability:ti) OR (generalisability:ti) OR (generalizability:ti) OR (adaptability:ti) OR (adaptation:ti) OR (transferability:ti) OR (portability:ti) OR (adoption:ti) OR (exchangeability:ti) OR (Toolkit*:ti) OR (Checklist*:ti))	**626**
**Cochrane Library**	((applicability):ti OR (generalisability):ti OR (generalizability):ti OR (adaptability):ti OR (adaptation):ti OR (transferability):ti OR (portability):ti OR (adoption):ti OR (exchangeability):ti OR (Toolkit*):ti OR (Checklist*):ti) AND	**271**

Data bases	Search strategy	NO. of documents
	((Technology Assessment Biomedical):ti,ab OR (health technology assessment):ti,ab OR (Cost-Benefit Analysis):ti,ab OR (economic evaluation analysis):ti,ab OR (cost-effectiveness Analysis):ti,ab OR (cost-utility Analysis):ti,ab OR (HTA):ti,ab)	
**epistemonikos**	(title:((title:(Technology Assessment Biomedical) OR abstract:(Technology Assessment Biomedical)) OR (title:(health technology assessment) OR abstract:(health technology assessment)) OR (title:(Cost-Benefit Analysis) OR abstract:(Cost-Benefit Analysis)) OR (title:(economic evaluation analysis) OR abstract:(economic evaluation analysis)) OR (title:(cost-effectiveness Analysis) OR abstract:(cost-effectiveness Analysis)) OR (title:(cost-utility Analysis) OR abstract:(cost-utility Analysis)) OR (title:(HTA) OR abstract:(HTA)))<br>AND<br>((title:(applicability) OR abstract:(applicability)) OR (title:(generalisability) OR abstract:(generalisability)) OR (title:(generalizability) OR abstract:(generalizability)) OR (title:(adaptability) OR abstract:(adaptability)) OR (title:(adaptation) OR abstract:(adaptation)) OR (title:(transferability) OR abstract:(transferability)) OR (title:(portability) OR abstract:(portability)) OR (title:(adoption) OR abstract:(adoption)) OR (title:(exchangeability) OR abstract:(exchangeability)) OR (title:(Toolkit) OR abstract:(Toolkit)) OR (title:(Checklist*) OR abstract:(Checklist*))))	**746**
Total	**10,375**	
(-duplicates)	**(-1,838)**	
**Final**	**8,537**	

Search strategies in all related health economic databases

Data bases	Search strategy	NO. of documents
Health Economics Evaluation Database (HEED)	(applicability) OR (generalisability) OR (generalizability) OR (adaptability) OR (adaptation) OR) ((transferability) OR (portability) OR (adoption) OR (exchangeability) OR (Toolkit) OR (Checklist)	**211**
EconLit	(applicability) OR (generalisability) OR (generalizability) OR (adaptability) OR (adaptation) OR ((transferability) OR (portability) OR (adoption) OR (exchangeability) OR (Toolkit) OR (Checklist)	**198**
Economic Working Paper	Abstract: (transferability) OR (generalisability) OR (generalizability)<br>AND<br>(Toolkit) OR (Checklist)	**989**
NHS Economic Evaluation Database	(applicability) OR (generalisability) OR (generalizability) OR (adaptability) OR (adaptation) OR ((transferability) OR (portability) OR (adoption) OR (exchangeability) OR (Toolkit) OR (Checklist)	**346**
Total	**1,744**	
(-duplicates)	**(-1,178)**	
**Final**	**566**	
